# Autism Screening in the First Year and Beyond: Comparison of Multiple Measures

**DOI:** 10.1002/aur.70340

**Published:** 2026-08-22

**Authors:** Devon N. Gangi, Chandni Parikh, Monique Moore Hill, Shyeena Maqbool, Rachel Ni, Simon Dvorak, Gregory S. Young, Sally Ozonoff

**Affiliations:** 1 MIND Institute, Department of Psychiatry and Behavioral Sciences, University of California-Davis, Sacramento, California, USA; 2 Rady Children’s Health, Autism Discovery Institute, San Diego, California, USA; 3 Information and Educational Technology, Enterprise Applications, University of California-Davis, Sacramento, California, USA

**Keywords:** autism, early detection, psychometric properties, screening

## Abstract

Autism screening aids in early detection and diagnosis. Several screeners are available for toddlers 16 months and older, but there is little data on the accuracy of screening measures used in the first year of life. A community sample (*N* = 1641) completed three screeners—M-CHAT-R, ITC, VIRSA—online at 6, 9, 12, 18, and 24 months of age (including exploratory use of M-CHAT-R prior to 16 months). At 36 months, outcomes of ASD or Non-ASD were determined by tele-evaluation and outcome surveys. Psychometric properties were assessed for all screeners at all ages, both used individually and in combination. When used in the first year of life (6–12 months), psychometric properties were in the poor range, with area under the curve (AUC) < 0.70 for all three screeners, whether used in isolation or combination. At 18 and 24 months, the M-CHAT-R and ITC outperformed the VIRSA, with psychometric properties in the fair range (AUC 0.70–0.79) used in isolation and the good range (AUC ≥ 0.80) when used in combination. Using the M-CHAT-R and ITC at more than one age in the second year also fell in the good range. This study, using procedures to address limitations of prior screening studies and follow screen-negative cases, found that none of the three screeners had adequate psychometric properties in the first year. At 18 and 24 months, findings supported the use of multiple screeners, either in combination or longitudinally, for best prediction of autism diagnosis.

## Summary

Autism screening helps to diagnose autism early in development. We found that the three screeners tested did not work well in the first year of life. At 18 and 24 months, using multiple screeners and screening at multiple ages worked best in predicting autism diagnosis.

Autism is characterized by challenges in social communication and the presence of restricted and repetitive behaviors (APA 2022). Screening tools aid in early detection of autism, which is crucial for access to early intervention services. Children with positive autism screening receive autism diagnoses earlier than those who are not screened or screen negative (Guthrie et al. 2019; Zhang et al. 2022).

Screening tools that could identify autism risk during infancy offer the opportunity for intervention before the full set of symptoms is present, but few are designed for infants in the first year of life, and their ability to identify autism at such young ages is not yet well-established (Sobieski et al. 2022; Zwaigenbaum et al. 2015). Previous studies of very early screening have primarily been conducted with 12-month-olds. The First Year Inventory (FYI; Baranek et al. 2003; Reznick et al. 2007), for example, was developed to assess autism risk at the first birthday. Preliminary psychometrics indicated that 44% of children with autism were identified by the FYI at 12 months (i.e., sensitivity), with a positive predictive value (PPV) of 0.14 (Turner-Brown et al. 2013). A newer short form (First Year Inventory—Lite Version; FYI-L) has shown impressive sensitivity of 0.67 at 12 months, but low PPV of 0.24, indicating a high false positive rate at this young age (Porto et al. 2025). The Infant-Toddler Checklist (ITC) provides normative data from 6 through 24 months (Wetherby et al. 2008). Pierce et al. (2011) found that approximately 20% of those who screened positive in a pediatric setting at 12 months were ultimately diagnosed with autism; more recent estimates of the ITC’s sensitivity and PPV at 12 months were also low, 0.26 and 0.24 respectively (Porto et al. 2025). As with the FYI, the ITC’s psychometric properties before 12 months have not yet been studied. Few other screeners have been developed for use earlier than 12 months and there is limited data on their psychometric properties (Petrocchi et al. 2020; Thabtah and Peebles 2019). The Autism Parent Screen for Infants (APSI; Sacrey et al. 2018) and the Parent Observation of Early Markers Scale (POEMS; Feldman et al. 2012) were developed for use with infants at elevated familial likelihood of autism, including ages prior to 12 months, but have not yet been studied in community samples.

The American Academy of Pediatrics recommends universal autism screening at 18 and 24 months, followed by referral for diagnostic evaluation when increased likelihood of developmental disorders is identified (Hyman et al. 2020). However, the US Preventive Services Task Force released a recommendation statement concluding that there is insufficient evidence to weigh the benefits and harms of universal autism screening, partly due to a lack of data on outcomes for children who screened negative (Siu et al. 2016). Most screening studies cannot calculate psychometric indices of sensitivity and specificity since they typically assess only children who have screened positive. This strategy is reasonable and more efficient in large samples where the majority of children will screen negative, but this does not allow for identification of cases of autism missed by screeners (Levy et al. 2020; McPheeters et al. 2016; Siu et al. 2016). Even when universal autism screening is implemented, there is evidence that many children with autism diagnoses in their medical records had screened negative (Guthrie et al. 2019), highlighting that follow-up of children who screened negative is crucial to obtaining accurate psychometric properties of screening instruments. A study that included diagnostic evaluations for children who screened positive *and* negative found lower sensitivity for the screener than reported in prior studies without diagnostic testing for screen-negative cases (Sturner et al. 2022). Recommendations for research on autism screening emphasize that accurate calculation of sensitivity and specificity requires following children who screen negative (Zwaigenbaum et al. 2015). One goal of the current investigation was to address this limitation of previous studies.

This study focuses on the psychometric properties of three autism screeners: the Modified Checklist for Autism in Toddlers, Revised (M-CHAT-R; Robins et al. 2014), the ITC (Wetherby et al. 2008), and the Video-Referenced Infant Rating System for Autism (VIRSA; Young et al. 2020). The M-CHAT-R was selected because it is the most widely used screener in pediatric settings. Though it was designed and validated for use between 16 and 30 months of age, we also administered the M-CHAT-R in the first year to explore the utility of using this screener earlier, as several items measure behaviors that emerge before 16 months (Inada et al. 2010). The ITC was selected as an instrument that is widely and freely available, brief, and provides norm-based standard scores as early as 6 months of age (Wetherby et al. 2008). The VIRSA was selected as an alternative to traditional paper-and-pencil screeners, instead providing video examples from which parents make selections. It was developed and validated for children 6 to 24 months of age (Young et al. 2020).

Previous estimates of the psychometric properties of these screeners are impacted by characteristics of the samples, including sample size, familial likelihood of autism, concurrent v. prospective case confirmation, and use of follow-up interviews. The M-CHAT-R is the most widely used screener in both research and primary care and has shown a range of sensitivity (22%–100%) and specificity (27%–100%) across studies, with a meta-analysis reporting a pooled sensitivity of 83% and a pooled specificity of 95% (Wieckowski et al. 2023). When implemented in a universal screening approach, sensitivity was lower (39%), with estimates higher in older toddlers and with more than one screening (Guthrie et al. 2019). The ITC is a caregiver-report questionnaire containing questions about social communication, language, and symbolic development that can be used to screen for autism. Wetherby et al. (2008) reported sensitivity of 93%, though children who screened negative were not systematically evaluated. The VIRSA is a screening tool that employs video examples representing a range of social communication ability rather than verbal descriptions. In a sample including children with elevated familial likelihood of autism who completed the VIRSA between 6 and 18 months of age, sensitivity ranged from 54%–78% and specificity ranged from 44%–77% (Young et al. 2020). However, the VIRSA has not yet been evaluated in a large, community-based sample.

In this study, we addressed limitations of prior research by using multiple simultaneous screening measures, conducting repeat screenings, and evaluating a random sample of children who screened negative. Our objectives were to evaluate the psychometric properties of the three autism screeners when used independently and in combination in a large community sample: (1) initially examining their performance in the first year of life (6, 9, and 12 months of age) and (2) then examining their psychometric properties at ages of common pediatric screening in the second year (18 and 24 months).

## 1 | Method

### 1.1 | Participants

In this longitudinal study, conducted between 2018 and 2024, three screeners for autism were administered online to caregivers of a sample of 2005 children. The sample was community-based (not enriched for autism) and participants were recruited via hospital records, state birth records, and social media. The vast majority of participants resided in California (94%). Families were eligible to participate if they had: (1) a child under the age of 10 months, (2) a valid email address, (3) access to a device with internet capacity, and (4) the ability to complete questionnaires at a 5th grade English reading level. Participants were not excluded based on any prior genetic or medical conditions, family history, or prematurity.

### 1.2 | Procedures

All study procedures were approved by the university’s Institutional Review Board. Caregivers completed screening instruments when participants were 6, 9, 12, 18, and 24 months of age. If a child screened positive on any screener administered between 6 and 24 months, they were invited for a diagnostic tele-assessment at 24 and 36 months of age (screening criteria for each measure are detailed below). Ten percent of children who screened negative on all measures at all ages (after inviting 15%) participated in tele-assessment at 24 and 36 months of age. Analyses used the 36-month tele-evaluation data, as this was considered the child’s final diagnostic outcome for the study.

Telehealth platforms were used to conduct diagnostic evaluations in this study for several reasons: (1) to permit data collection during COVID-19 restrictions, (2) to allow participation for families residing far from the study site, and (3) to accommodate the large number of assessments conducted. Tele-assessments were completed by Masters- or PhD-level examiners with specialized training in both autism evaluations and tele-assessment methods, with direct supervision by a licensed clinical psychologist. Examiners were unaware of participants’ screening status or results of prior evaluations. Telehealth evaluations included caregiver report of developmental functioning using the Developmental Profile, 4th Edition (DP-4; Alpern 2020), observation of behaviors associated with autism using the TELE-ASD-PEDS (TAP; Corona et al. 2020), and clinical interviewing about autism-related characteristics and behaviors as necessary. Telehealth evaluation using the DP-4 and TAP has strong caregiver satisfaction and high rates of agreement (over 90%) with in-person evaluation (Gangi et al. 2025; Ozonoff et al. 2025; Parikh et al. 2025). At the end of the tele-evaluation, examiners classified children as meeting *DSM-5* criteria (APA 2022) for autism spectrum disorder (ASD) or not based on all information collected (see below). In rare cases when tele-assessment was inconclusive (*n* = 7, 1%), families completed in-person evaluation for diagnostic clarification.

**FIGURE 1 |:**
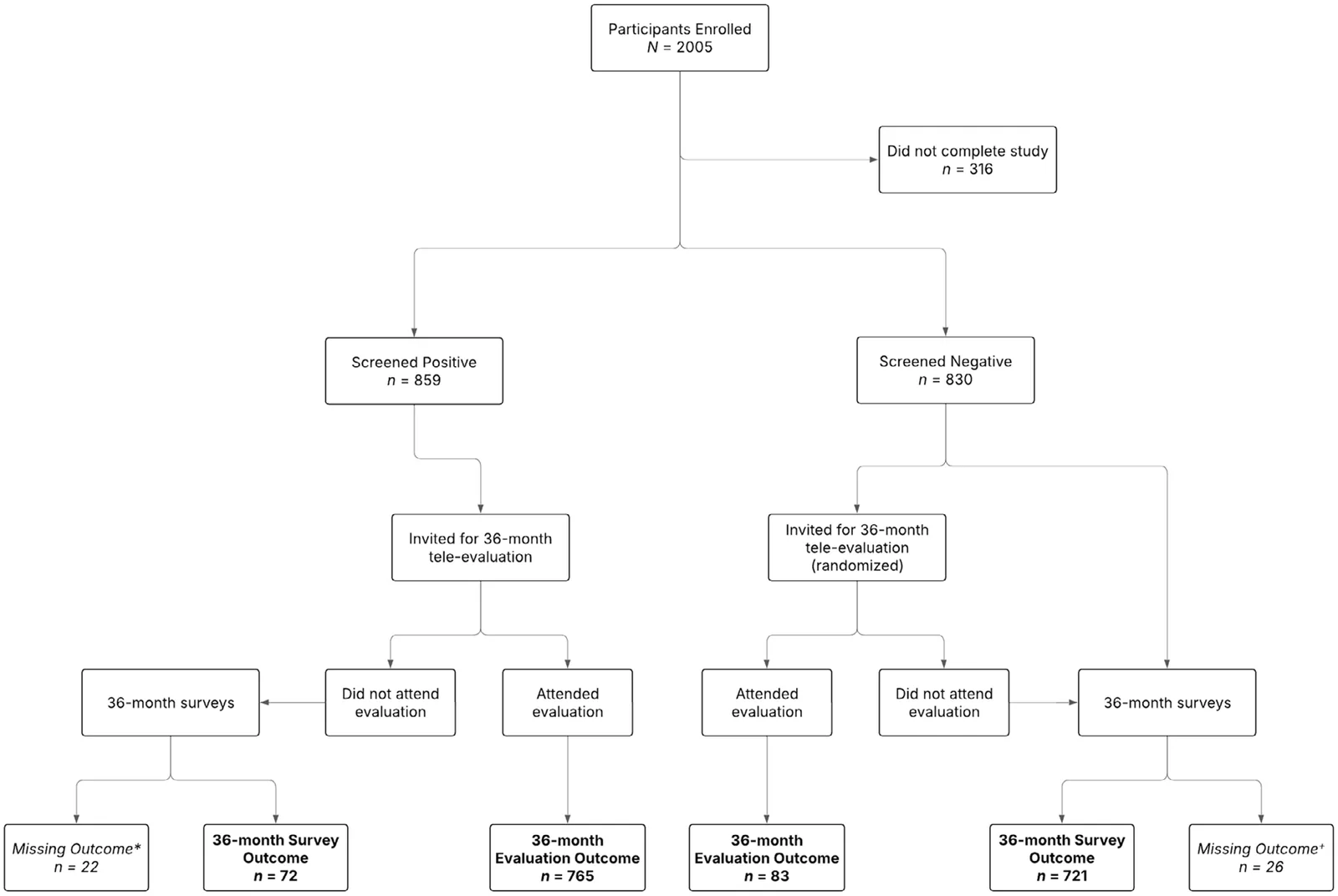
Flowchart of longitudinal study flow for participants. *Note:* Screening status reflects *overall* study screening status (i.e., screen positive = positive screen on any screener at any age; screen negative = screened negative on all screeners completed). * < 2 36-month measures (*n* = 7); ≥ 1 36-month measure elevated (*n* = 12); indicated outside autism diagnosis, unverified (*n* = 3). ^+^ < 2 36-month measures (*n* = 20); indicated outside autism diagnosis, unverified (*n* = 6).

At 36 months, three outcome surveys were completed by all participating families, whether they had done tele-assessments or not. These surveys measured autism characteristics and conducted surveillance for autism diagnoses made outside the study. At the conclusion of the study, when participants were between 3 and 7 years of age, depending on time of enrollment, all participating families were surveyed again about outside diagnoses of ASD. Families were included in analyses if they had at least one screener completed at any age and study outcome determination based on either tele-assessment or surveys (see below). Figure 1 depicts longitudinal study flow for participants.

### 1.3 | Measures

#### 1.3.1 | Online Screening Measures Administered at 6, 9, 12, 18, and 24 Months of Age

##### 1.3.1.1 | Modified Checklist for Autism in Toddlers, Revised (M-CHAT-R).

The M-CHAT-R is a caregiver-report checklist with 20 yes/no items. It was initially validated for use with children 16 to 30 months of age but is often used up to 48 months (Wieckowski et al. 2023). A child was considered to screen positive on the M-CHAT-R if their initial score was ≥ 3 at 18 or 24 months of age. For children who scored above the cutoff, families were contacted to conduct the M-CHAT-R’s follow-up interview, which is recommended clinically to reduce false-positive identifications. However, the follow-up score was not used to determine screen-positive status or in analyses, by design, since one objective of this study was to cast as wide a net as possible to identify autism cases that might be missed through traditional screening procedures. Thus, children whose *initial* M-CHAT-R scores were ≥ 3 were considered screen-positive for study purposes and invited for evaluation regardless of follow-up score.

Inada et al. (2010) published passage rates of each M-CHAT-R item in a general population sample aged 8–20 months. They found that many M-CHAT-R items were passed by 75% or more of community infants at ages lower than 16 months (the current recommended age of use). Specifically, 14 of 20 M-CHAT-R items were passed by at least 75% of infants below 16 months of age and 12 items were passed by at least 75% of infants 12 months or younger. Given the potential developmental appropriateness of many items, we had parents complete the M-CHAT-R at all screening points (6–24 months) in order to explore its utility at younger ages. For exploratory purposes, the M-CHAT-R from the first year (6, 9, and 12 months) was scored using the same procedure as older ages—i.e., initial scores ≥ 3 were considered screen positives.

##### 1.3.1.2 | Infant-Toddler Checklist (ITC).

The ITC is a caregiver-report questionnaire about social communication, language, and symbolic development, normed for use between 6 and 24 months of age. It yields three composite scores (Social, Speech, Symbolic) and a total score. A child was considered to screen positive on the ITC if their composite and/or total scores were below the 10th percentile at any age. The ITC was added to the study protocol partway through data collection, so it is missing for more participants than other screeners.

##### 1.3.1.3 | Video-Referenced Infant Rating System for Autism (VIRSA).

The VIRSA is a video-based screening tool using videos of parents and infants playing together, depicting a range of social communication ability (ranging from 1 = least social to 10 = most social). Pairs of videos are presented to caregivers, and they select which video is most similar to how their child behaves. We hypothesized that the VIRSA could improve screening by using video examples for parents to rate, rather than written descriptions of behavior. A child was considered to screen positive if their score was ≤ 3, the range of concern identified in Young et al. (2020).

#### 1.3.2 | 36-Month Survey Outcome Measures

Three questionnaires were administered to all families at 36 months to identify any autism cases that might have been missed by the 6- to 24-month screenings and to determine diagnostic outcome for participants who had not screened positive on any measure and/or had not attended a diagnostic tele-assessment. If 36-month questionnaires indicated any autism concerns or a diagnosis made outside the study, the participant was invited for tele-assessment to clarify outcome classification.

##### 1.3.2.1 | Social Responsiveness Scale, Second Edition (SRS-2).

The SRS-2 is a caregiver-report questionnaire that captures social skills and behavior associated with autism (Constantino and Guber 2012). It is normed for children 2–18 years of age. Total scores ≥ 60 indicated potential concerns for autism.

##### 1.3.2.2 | Modified Checklist for Autism in Toddlers, Revised (M-CHAT-R).

This instrument was re-administered at 36 months, not as a screener but as an additional measure of autism symptoms at study outcome. The cutoff used was again an initial score of ≥ 3 (Robins et al. 2014).

##### 1.3.2.3 | Study Exit Survey.

At both 36 months and at the conclusion of study funding (approximately 6 years after enrollment of initial participants), all families were contacted and asked to complete a surveillance survey regarding whether their child had received a diagnosis of autism outside of the study, by whom, and at what age.

#### 1.3.3 | Outcome Determination

Two methods were used to assign a binary diagnostic status (ASD, Non-ASD) at study outcome, based on either tele-assessment (“Evaluation Outcome”) or 36-month survey outcome measures (“Survey Outcome”) (see Figure 1). For participants seen for a diagnostic tele-assessment at 36 months (*n* = 848; mean age = 35.18, SD = 2.07), the result of the evaluation (ASD or Non-ASD) was used as their diagnostic outcome. For children who did not complete a 36-month diagnostic tele-evaluation, primarily screen-negative cases, outcomes were determined using the survey outcome measures. Before assigning a Non-ASD outcome classification based on surveys, however, we attempted to identify additional false-negative cases that may have slipped through earlier screenings. For additional outcome certainty for the children who were not seen for a tele-assessment, we required that no autism concerns were raised on any of the survey outcome measures completed by caregivers at 36 months: the M-CHAT-R (i.e., score < 3), SRS (i.e., score < 60), and Study Exit Survey (i.e., no outside autism diagnosis reported). If children had at least 2 of these 3 measures and all of them indicated no concern for autism, their study outcome was classified as Non-ASD. Of participants without a tele-assessment who completed the study, 793 were given Non-ASD survey outcomes. Children missing outcomes either (1) did not complete the study (*n* = 317), (2) had fewer than two 36-month measures completed (*n* = 27), (3) had ≥ 1 36-month measure elevated for autism concern (*n* = 12) and were invited but not seen for tele-assessment to clarify diagnostic status, or (4) indicated an outside autism diagnosis that was not able to be verified by the study (*n* = 9). This procedure resulted in a final sample of 1641 participants with study outcome determinations (99 children with ASD outcomes made by the study team and 1542 children with Non-ASD outcomes) who were included in the present analyses. Demographic information for each outcome group is presented in Table 1.

### 1.4 | Analytic Plan

We used ROC analysis to assess sensitivity, specificity, PPV, and negative predictive value (NPV) of the screeners in predicting autism outcome. Area under the curve (AUC) was computed as a measure of the ability to distinguish between groups and overall diagnostic performance. AUC values of ≥ 0.90 fall in the excellent range of discrimination, 0.80–0.89 in the good range, 0.70–0.79 in the fair range, 0.60–0.69 in the poor range, and ≤ 0.60 in the failing range (with values around 0.50 indicating chance level discrimination) (Çorbacıoğlu and Aksel 2023; Nahm 2022). This was conducted first for each screener in isolation and in combination, across all ages, then longitudinally in the second year of life (18 and 24 months). We then conducted logistic regressions and correlations to examine the unique predictive value of each measure, above and beyond the others.

## 2 | Results

### 2.1 | Psychometric Properties of Screeners Used in Isolation

#### 2.1.1 | First Year of Life

Psychometric properties for each screener were first assessed in isolation (see Table 2). When used in the first year of life (6, 9, and 12 months), the range of AUC values across all screeners was 0.49 to 0.65 (poor or failing range). The VIRSA tended to have low sensitivity (12%–24%), but high specificity (93%–95%). The ITC also tended to have lower sensitivity (31%–53% across ages) than specificity (71%–90%). The M-CHAT-R, using the standard cutoff of 3, resulted in very high screen-positive rates in the first year of life. This resulted in sensitivity rates that were extremely high (89%–99% across ages), but specificity rates that were very low (< 1%–33%).

#### 2.1.2 | Second Year of Life

At the common ages of pediatric screening—18 and 24 months—the AUC values for screeners used in isolation fell within the poor range for the VIRSA (0.65 at both ages) and the fair range for the ITC and M-CHAT-R (0.74–0.78 across instruments and ages; see Table 2). As in the first year of life, the VIRSA continued to have low sensitivity (33%–38%), but high specificity (92%–97%). The ITC and M-CHAT-R had improved sensitivity (53%–63%) relative to the first year of life and high specificity (90%–97%).

### 2.2 | Psychometric Properties of Screeners Used in Combination

We next examined the psychometric properties of the three screeners used together in various combinations at a single age (see Table 3). We used two scoring rules to define screening positive on a combination of instruments: the narrow definition required a positive screen on *both* of 2 screeners or *all* of 3 screeners within the combination, while the broader definition allowed a positive screen on *either* of 2 screeners or *any* of 3 screeners. ROC analysis was again used to assess sensitivity, specificity, PPV, and NPV in predicting ASD outcome for each combination at each age (see Table 3).

#### 2.2.1 | First Year of Life

AUC remained in the poor to failing range (Çorbacıoğlu and Aksel 2023; Nahm 2022), 0.49–0.69 for every combination of screeners used in the first year of life (6, 9, and 12 months). It is worth noting that due to the extremely high screen positive rate of the M-CHAT-R in the first year, any combination that included the M-CHAT-R resulted in high sensitivity (89%–99%) but very low specificity (< 1%–31%).

#### 2.2.2 | Second Year of Life

Psychometric properties for combinations of screeners were improved at the common ages of pediatric screening relative to using any instrument alone. For example, at 18 months, AUC improved to the fair range for several screener combinations (see Table 3). At 24 months, AUC improved still more, with many screener combinations in the good range (above 0.80). At both ages, narrower definitions requiring a positive screen on *both* or *all* screeners performed poorly. The best performance was achieved using broader decision rules allowing a positive screen on *either* or *any* instrument in the combination at 24 months, with all AUCs in the good range, sensitivity ranging from 66% to 78%, and specificity ranging from 92% to 94%.

### 2.3 | Psychometric Properties of Screeners Incorporating Multiple Ages in the Second Year of Life

We also examined whether longitudinal screening in the second year of life improved autism prediction, first using the same instrument longitudinally and then using multiple instruments over time. Given the poor performance of all screeners in the first year of life, these analyses were restricted to the second year of life; similarly, the VIRSA was not included in these analyses due to low performance at all ages. As was the case for using combinations of screeners at a single age, using the same screener at multiple ages resulted in better psychometric properties for both the M-CHAT-R and the ITC when using a broader (“or”) definition than a narrower (“and”) definition of screen-positive status. That is, for both instruments, requiring a positive screen at *either* age (18 *or* 24 months) performed better than requiring a positive screen at *both* ages (see Table 4). AUC improved from the poor to fair range when requiring a positive screen at *both* ages (0.71 and 0.69 for the M-CHAT-R and ITC, respectively) to the good range when using a positive screen at *either* age (0.81 and 0.83 for the M-CHAT-R and ITC, respectively). Sensitivity also improved, from 39%–43% (narrow definition requiring a positive screen at both ages) to 73%–75% (broad definition allowing a positive screen at either age).

When using the two screeners—M-CHAT-R and ITC—in combination at both ages, we found a similar pattern. As can be seen in Table 4, defining a positive screen on either measure at *either* age (18 *or* 24 months) resulted in better psychometric properties (AUC = 0.85, sensitivity 88%) than requiring a positive screen on either measure at *both* ages (AUC = 0.74, sensitivity 51%).

### 2.4 | Unique Predictive Value of Screeners

Finally, we conducted logistic regressions to examine the unique predictive value of each measure, above and beyond the others. Given the poor performance of all screeners in the first year of life, these analyses were completed only for the second year of life. Final model parameters are presented in Table 5. At 18 months, a model with only the M-CHAT-R predicting ASD outcome was a significant improvement over an empty model, *χ*^2^(1) = 38.15, *p* < 0.001. Adding the ITC significantly improved prediction, *χ*^2^(1) = 49.20, *p* < 0.001, and further adding the VIRSA again significantly improved prediction, *χ*^2^(1) = 46.50, *p* < 0.001. At 24 months, a model with only the M-CHAT-R predicting ASD outcome was a significant improvement over an empty model, *χ*^2^(1) = 62.44, *p* < 0.001. Adding the ITC significantly improved prediction, *χ*^2^(1) = 69.27, *p* < 0.001, and further adding the VIRSA again significantly improved prediction, *χ*^2^(1) = 54.63, *p* < 0.001. This indicates that at both 18 and 24 months, all three screeners were uniquely predictive and, even with all three in the model, each remained significant (see Table 5).

Correlations between measures found that at both 18 and 24 months, the VIRSA was weakly correlated with the M-CHAT-R and ITC, *r*s = 0.21–0.28, *p*s<0.001. The M-CHAT-R and ITC were moderately correlated at 18 months, *r* = 0.57, *p* < 0.001, and 24 months, *r* = 0.62, *p* < 0.001. These intercorrelations further support that the measures do not fully overlap and provide unique contributions.

## 3 | Discussion

This study evaluated the psychometric characteristics of three autism screeners in the first year of life (6, 9, and 12 months) and at common ages of pediatric screening in the second year of life (18 and 24 months). Multiple screeners were completed at multiple ages on a large sample, diagnostic follow-up was conducted on a sub-sample of randomly selected screen-negative cases, and additional surveillance was done at the end of the study to capture outside diagnoses made after study participation was complete. This permitted us to identify many children who would have been screen-negatives if only one screener had been used, screening had been done at only one age, or surveillance after age 3 had not been conducted. Identifying many (although likely not all) false negatives provides more accurate estimates of sensitivity and specificity measurement than previous screening studies, which typically do not evaluate screen-negatives to find missed cases.

### 3.1 | Screening in the First Year of Life

In the first year of life, all three screeners—VIRSA, ITC, and M-CHAT-R—performed poorly at all ages. The AUC, a summary metric reflecting a measure’s ability to distinguish between outcome groups (Çorbacıoğlu and Aksel 2023; Nahm 2022), was in the failing (i.e., below 0.60) to poor range (i.e., between 0.60 and 0.69) for all measures, whether used individually or in combination. The M-CHAT-R was ineffective in the first year of life. The vast majority of children screened positive at these ages, resulting in AUCs in the failing range. This is not surprising since the M-CHAT-R’s cutoff of 3 was validated for children 16 months and older.

While the VIRSA’s AUC values were also in the failing range, this instrument did not suffer from elevated screen positive rates like the M-CHAT-R, but instead tended toward under-identification, with low sensitivity rates in the first year (12%–24%). This is consistent with a prior study of the VIRSA, in which AUCs in the first year of life also fell in the failing range in a sample of children at elevated familial likelihood of autism (Young et al. 2020).

The ITC, which was normed for infants in the first year of life, performed only slightly better than the M-CHAT-R and VIRSA, with AUCs in the poor (as compared to the failing) range. Wetherby et al. (2008), the developers of the ITC, also found low sensitivity under 12 months of age in detecting autism.

Taken together, these findings do not offer promise for screening for autism in the first year of life *using the currently investigated measures and scoring cutoffs*. As noted, this was not unexpected for the M-CHAT-R, which is recommended for 16 months and older. We administered it at younger ages in an exploratory fashion, given the potential developmental relevance of many items (Inada et al. 2010), but it did not fare well in the first year of life. The other two screeners employed in this study were explicitly developed for use in the first year of life, yet neither performed satisfactorily in this age range either. The ITC’s norms go down to 6 months and the VIRSA was developed using videos of infants demonstrating autistic features at 6, 9, and 12 months, but both had poor psychometric properties when used in the first year of life as well. The poor performance of all three screeners at 12 months and before may be due to the timing of symptom onset, with autistic features emerging gradually over time (Landa et al. 2007; Ozonoff et al. 2010; Szatmari et al. 2016). It is possible that in the first year of life, clearly atypical or delayed behavior is not yet readily observable for caregivers to rate consistently.

Before a determination can be made about the validity of autism screening prior to the first birthday, further studies are needed to test other instruments that are developmentally appropriate in this age range. For example, the recently updated First Year Inventory, version 3.1 (Baranek et al. 2022) extends down to 6 months, and both the APSI (Sacrey et al. 2018) and POEMS (Feldman et al. 2012) have been used prior to the first birthday. Another potentially fruitful avenue for future research is multi-stage longitudinal screening that begins in the first year of life. One study demonstrated that using the ITC or FYI at 12 months, combined with the M-CHAT-R at ages after 16 months, led to earlier autism diagnosis (Wieckowski et al. 2021); future research could further investigate this approach using additional screening instruments.

### 3.2 | Common Ages of Pediatric Screening in the Second Year of Life

When considering the three screeners used in isolation, the ITC and M-CHAT-R both clearly outperformed the VIRSA at 18 and 24 months. The AUC in the second year of life was in the poor range (i.e., between 0.60 and 0.69) for the VIRSA, while it was in the fair range (i.e., between 0.70 and 0.79) for both the ITC and M-CHAT-R. Therefore, the results of this study do not support further use or development of the video-based VIRSA, which did not enhance identification of autism above traditional written behavioral descriptions of screeners.

We also evaluated psychometric properties when screeners were used in combination. In general, requiring a positive screen on multiple instruments (using a narrower definition; “and” rule) resulted in low sensitivity at any age (24%–51%). Performance was improved, however, when defining a positive screen as over the cutoff on *either* of two instruments or *any* of three instruments (broader definition; “or” rule). This provided a better balance between sensitivity and specificity. AUC values for M-CHAT-*R* + ITC combinations at 18 months fell in the fair range, and at 24 months fell in the good range, considered clinically useful (i.e., between 0.80 and 0.90) (Çorbacıoğlu and Aksel 2023; Nahm 2022). Similarly, at both 18 and 24 months, logistic regression demonstrated that each screener provided non-redundant information that identified true positive cases of autism that would not have been detected by the other screeners (i.e., would have been false negatives). Taken together, the clinical implication of these findings is that sensitivity to detect autism, while maintaining acceptable specificity, is maximized by using two screeners together at 18 and 24 months.

We found better performance of autism screeners at 24 months than 18 months of age. This is consistent with previous work using the M-CHAT-R/F finding that screenings at older ages (e.g., 21–26 months of age) are more sensitive and have higher PPV than those conducted at younger ages (e.g., 16–20 months of age) (Guthrie et al. 2019; Sturner et al. 2017). Conducting repeated screenings identifies additional children with autism even when initial screening is negative (Dai et al. 2020; Parikh and Ozonoff 2025; Wieckowski et al. 2023). There have also been suggestions to use an early screener such as the ITC or FYI at 12 months, followed by the M-CHAT-R at 16 months and above (Salgado-Cacho et al. 2021; Wieckowski et al. 2021). In our sample, for both the M-CHAT-R and the ITC, considering a positive screen at *either* 18 or 24 months yielded higher AUC values than for either age independently. This fits with what is known about the emergence of autism features in the first two years of life, namely that it is gradual and its time course differs across children, with some showing clear signs early and others not until later (Landa et al. 2007; Ozonoff et al. 2010; Szatmari et al. 2016). Even when children are followed closely by experts conducting repeated developmental evaluations, many children with autism outcomes at 36 months are not yet diagnosed (or diagnoseable) at 18 or 24 months (Ozonoff et al. 2015). As features of autism emerge over time, with children meeting criteria for a diagnosis at different times in development, repeated screenings—even after an early negative screening—minimize the likelihood of missing children who would benefit from further evaluation.

### 3.3 | Study Strengths and Limitations

Our study design allowed for the calculation of all psychometric performance metrics (not only PPV and NPV, but also sensitivity and specificity). Multiple screeners were administered at multiple ages, and any child who screened positive on *any* measure at *any* age was invited for evaluation. For example, a child who screened positive on one screener at one age (e.g., a positive screen on the VIRSA at 6 months) was invited for evaluation even if all other screeners at all other ages were negative. This resulted in over half our sample being seen for diagnostic evaluation, including many children who would have been considered screen-negatives on individual measures or single ages. Conducting surveillance at the end of the study also allowed for greater confidence in presumed Non-ASD outcomes. Rather than relying on negative screens alone, children were only classified as Non-ASD if there were no concerns for autism on additional survey outcome measures.

The autism rate in our sample was 6%. Although this is higher than national prevalence estimates of 3% among 4-year-old children, it is consistent with the most recent estimates of the autism rate in California among 4-year-olds, which is also 6% (Shaw et al. 2025). The vast majority of our sample (94%) resides in California, a state that has consistently had the highest prevalence among sites of the Autism and Developmental Disabilities Monitoring Network (Shaw et al. 2025). Additionally, while we did not enrich our community sample for autism risk, we also did not exclude participants with genetic/medical diagnoses that could affect neurodevelopment (e.g., hydrocephalus, Down syndrome) or who had older siblings with autism, since these would be expected to occur in a general community sample. The rates of each were low (genetic/medical conditions = 1%, elevated familial likelihood = 4%) but may also have elevated the rate of autism in our sample.

A possible limitation of our study related to the M-CHAT-R is the use of the initial, rather than the follow-up, score. This was done by design, since one objective of this study was to identify as many false negatives as possible. However, previous studies show improved psychometric performance of the M-CHAT-R with use of the follow-up interview (e.g., Kleinman et al. 2008; Robins et al. 2014), and therefore it is possible that psychometric estimates in our sample might have been higher had we used the follow-up interview to define screen positivity on the M-CHAT-R. But while this might improve specificity (by decreasing false positives), it would not improve sensitivity (as the follow-up interview does not identify new cases).

### 3.4 | Clinical Implications

Our findings have clear applications for clinical practice. First, this study does not support autism screening in the first year of life using the three measures tested (M-CHAT-R, ITC, VIRSA) and currently available scoring criteria. It is not surprising to find that screening for autism in the first year of life is difficult, given that autism symptoms emerge over time and typically begin to be evident during the second year of life. Even studies with expert evaluations conducted systematically and prospectively (Zwaigenbaum et al. 2016) have not led to broad identification of children with autism during the first year of life, and often a child’s presentation is not clear enough for a diagnosis to be made until 24 or 36 months (Ozonoff et al. 2015).

Second, our findings support the use of multiple screeners at 18 and 24 months, particularly the two paper-and-pencil caregiver-report instruments already used in primary care, the M-CHAT-R and ITC. At both ages, these performed best when screening positive was considered screening positive on *either* instrument or at *either* age. This approach outperformed any of the screeners considered in isolation. It is possible that additional steps between initial screening and diagnostic evaluations may aid in triaging children to receive full diagnostic evaluations who may be most likely to benefit after an initial positive screen. For example, employing a Level 2 screener such as the Screening Tool for Autism in Toddlers and Young Children (STAT; Stone and Ousley 2008) after the M-CHAT-R can aid in reducing false positives and streamlining referrals for further autism-specific evaluation (Khowaja et al. 2018) or be used to determine presumptive eligibility for early intervention services while waiting for formal assessment and diagnosis (Rotholz et al. 2017).

While our suggested approach to screening maximizes the likelihood of identifying autism, there will also be false positives referred for evaluation. We acknowledge that this may contribute to a burdened system with limited providers in which families often wait long periods for a developmental evaluation, and it may be more difficult for children to be evaluated quickly outside the context of our research study. However, at the stage of initial autism screening, our findings clearly support the use of multiple screeners, with repeated screenings conducted in the second year of life, to maximize the likelihood of identifying autism and minimize the likelihood of children getting missed for evaluation during a crucial period for early intervention services.

## Figures and Tables

**TABLE 1 |:** Participant characteristics by diagnostic outcome group.

	ASD		Non-ASD	
	*n*	%	*n*	%
*Child sex*				
Male	74	75%	809	53%
Female	25	25%	733	48%
*Child ethnicity*				
Hispanic/Latino	30	30%	363	24%
Non-Hispanic/Latino	67	68%	1163	75%
Not reported	2	2%	16	1%
*Child race*				
American Indian/Alaska Native	0	0%	13	1%
Asian	14	14%	131	9%
Black/African-American	7	7%	33	2%
Native Hawaiian/Other Pacific Islander	0	0%	5	1%
White	36	36%	939	61%
More than one race	41	41%	407	26%
Not reported	1	1%	14	1%
*Maternal education*				
High school or less	10	10%	59	4%
Some college	25	25%	223	14%
Bachelor’s degree	30	30%	602	39%
Graduate degree	34	34%	657	43%
Not reported	0	0%	1	1%
*Annual household income*				
$25,000 or less	9	9%	24	2%
$25,001-50,000	18	18%	77	5%
$50,001-80,000	17	17%	240	16%
$80,001-100,000	7	7%	173	11%
$100,001-125,000	8	8%	199	13%
$125,001–150,000	7	7%	184	12%
$150,001-200,000	15	15%	219	14%
>$200,000	12	12%	321	21%
Not reported	6	6%	105	7%
*Familial likelihood of autism*				
Elevated Likelihood (older sibling with autism)	17	17%	47	3%
Typical Likelihood (no older sibling with autism)	82	83%	1495	97%
*Gestational age*				
≤ 32 weeks	2	2%	14	1%
33–36 weeks	8	8%	109	7%
> 36 weeks	89	90%	1419	92%
*Genetic/medical conditions*				
Caregiver-reported conditions	2	2%	8	1%

*Note:* Percentages may not add to 100 due to rounding.

**TABLE 2 |:** Psychometric properties for each measure used individually at each age.

	Total (*n*)	ASD outcome		Non-ASD outcome		Sensitivity	Specificity	Positive predictive value	Negative predictive value	Area under the curve	95% confidence interval
		Screen positive (true positives)	Screen negative (false negatives)	Screen positive (false positives)	Screen negative (true negatives)						
*6 months*											
VIRSA	1512	10	71	68	1363	12%	95%	13%	95%	0.54	0.47–0.61
ITC	1390	39	35	385	931	53%	71%	9%	96%	0.62	0.55–0.69
M-CHAT-R	1515	78	1	1430	6	99%	< 1%	5%	86%	0.50	0.43–0.56
*9 months*											
VIRSA	1535	10	71	78	1376	12%	95%	11%	95%	0.54	0.47–0.60
ITC	1387	23	51	131	1182	31%	90%	15%	96%	0.61	0.53–0.68
M-CHAT-R	1557	77	4	1436	40	95%	3%	5%	91%	0.49	0.42–0.56
*12 months*											
VIRSA	1558	19	62	99	1378	24%	93%	10%	96%	0.58	0.51–0.65
ITC	1385	31	44	143	1167	41%	89%	18%	96%	0.65	0.58–0.73
M-CHAT-R	1570	74	9	1003	484	89%	33%	7%	98%	0.61	0.55–0.66
*18 months*											
VIRSA	1593	36	59	117	1381	38%	92%	24%	96%	0.65	0.59–0.72
ITC	1404	47	39	96	1222	55%	93%	33%	97%	0.74	0.67–0.80
M-CHAT-R	1597	60	35	150	1352	63%	90%	29%	97%	0.77	0.71–0.83
*24 months*											
VIRSA	1530	29	60	45	1396	33%	97%	39%	96%	0.65	0.58–0.72
ITC	1484	54	36	61	1333	60%	96%	47%	97%	0.78	0.72–0.84
M-CHAT-R	1572	49	44	43	1436	53%	97%	53%	97%	0.75	0.68–0.81

**TABLE 3 |:** Psychometric properties of screeners used in combination at each age.

	Total (*n*)	ASD Outcome		Non-ASD Outcome		Sensitivity	Specificity	Positive predictive value	Negative predictive value	Area under the curve	95% confidence interval
		Screen positive (true positives)	Screen negative (false negatives)	Screen positive (false positives)	Screen negative (true negatives)						
*6 months*											
ITC + VIRSA	1374	6	68	26	1274	8%	98%	19%	95%	0.53	0.46–0.60
ITC or VIRSA	1379	43	32	427	877	57%	67%	9%	96%	0.62	0.56–0.69
ITC+M-CHAT-R	1387	39	35	385	928	53%	71%	9%	96%	0.62	0.55–0.69
ITC or M-CHAT-R	1514	78	1	1430	5	99%	< 1%	5%	83%	0.50	0.43–0.56
VIRSA + M-CHAT-R	1501	10	69	68	1354	13%	95%	13%	95%	0.54	0.47–0.61
VIRSA or M-CHAT-R	1515	78	1	1430	6	99%	< 1%	5%	86%	0.50	0.43–0.56
VIRSA + ITC+M-CHAT-R	1373	6	68	26	1273	8%	98%	19%	95%	0.53	0.46–0.60
VIRSA or ITC or M-CHAT-R	1514	78	1	1430	5	99%	< 1%	5%	83%	0.50	0.43–0.56
*9 months*											
ITC + VIRSA	1363	3	71	13	1276	4%	99%	19%	95%	0.51	0.45–0.59
ITC or VIRSA	1375	30	48	196	1101	39%	85%	13%	96%	0.62	0.55–0.69
ITC+M-CHAT-R	1386	23	51	131	1181	31%	90%	15%	96%	0.61	0.53–0.68
ITC or M-CHAT-R	1550	77	4	1436	33	95%	2%	5%	89%	0.49	0.42–0.55
VIRSA + M-CHAT-R	1532	10	71	76	1375	12%	95%	12%	95%	0.54	0.47–0.60
VIRSA or M-CHAT-R	1555	77	4	1438	36	95%	2%	5%	90%	0.49	0.42–0.55
VIRSA + ITC + M-CHAT-R	1363	3	71	13	1276	4%	99%	19%	95%	0.52	0.45–0.59
VIRSA or ITC or M-CHAT-R	1548	77	4	1438	29	95%	2%	5%	88%	0.49	0.42–0.55
*12 months*											
ITC + VIRSA	1372	10	63	28	1271	14%	98%	26%	95%	0.56	0.48–0.63
ITC or VIRSA	1382	40	35	214	1093	53%	84%	16%	97%	0.69	0.62–0.75
ITC+M-CHAT-R	1385	31	44	143	1167	41%	89%	18%	96%	0.65	0.58–0.73
ITC or M-CHAT-R	1502	74	9	1003	416	89%	29%	7%	98%	0.59	0.54–0.65
VIRSA + M-CHAT-R	1554	19	62	88	1385	24%	94%	18%	96%	0.59	0.52–0.66
VIRSA or M-CHAT-R	1562	74	9	1014	465	89%	31%	68%	98%	0.60	0.55–0.66
VIRSA + ITC + M-CHAT-R	1372	10	63	28	1271	14%	98%	26%	95%	0.56	0.48–0.63
VIRSA or ITC or M-CHAT-R	1498	74	9	1014	401	89%	28%	68%	98%	0.59	0.53–0.64
*18 months*											
ITC + VIRSA	1396	23	62	21	1290	27%	98%	52%	95%	0.63	0.56–0.70
ITC or VIRSA	1418	60	29	192	1137	67%	86%	24%	98%	0.77	0.71–0.82
ITC+M-CHAT-R	1403	44	42	57	1260	51%	96%	44%	97%	0.73	0.67–0.80
ITC or M-CHAT-R	1419	63	26	189	1141	71%	86%	25%	98%	0.78	0.73–0.84
VIRSA + M-CHAT-R	1588	28	66	28	1466	30%	98%	50%	96%	0.64	0.57–0.71
VIRSA or M-CHAT-R	1588	68	26	239	1255	72%	84%	22%	98%	0.78	0.73–0.84
VIRSA + ITC + M-CHAT-R	1395	23	62	13	1297	27%	99%	64%	95%	0.63	0.56–0.70
VIRSA or ITC or M-CHAT-R	1428	71	19	270	1068	79%	80%	21%	98%	0.79	0.74–0.84
*24 months*											
ITC + VIRSA	1440	20	65	9	1346	24%	99%	69%	95%	0.61	0.54–0.69
ITC or VIRSA	1448	63	25	97	1263	72%	93%	39%	98%	0.82	0.77–0.88
ITC+M-CHAT-R	1484	41	49	24	1370	46%	98%	63%	97%	0.72	0.65–0.79
ITC or M-CHAT-R	1486	62	28	80	1316	69%	94%	44%	98%	0.82	0.76–0.87
VIRSA + M-CHAT-R	1525	18	70	8	1429	21%	99%	69%	95%	0.60	0.53–0.67
VIRSA or M-CHAT-R	1530	60	31	80	1359	66%	94%	43%	98%	0.80	0.74–0.86
VIRSA + ITC + M-CHAT-R	1440	17	68	6	1349	20%	> 99%	74%	95%	0.60	0.53–0.67
VIRSA or ITC or M-CHAT-R	1452	70	20	114	1248	78%	92%	38%	98%	0.85	0.80–0.90

**TABLE 4 |:** Psychometric properties for screeners used longitudinally (18 and 24 months).

	Total (*n*)	ASD Outcome		Non-ASD Outcome		Sensitivity	Specificity	Positive Predictive Value	Negative Predictive Value	Area Under the Curve	95% Confidence Interval
		Screen Positive (True Positives)	Screen Negative (False Negatives)	Screen Positive (False Positives)	Screen Negative (True Negatives)						
*Individual screeners*											
M-CHAT-R											
18 months + 24 months	1549	39	52	26	1432	43%	98%	60%	96%	0.71	0.64–0.77
18 months or 24 months	1560	70	26	167	1297	73%	89%	30%	98%	0.81	0.76–0.86
ITC											
18 months + 24 months	1514	34	53	24	1403	39%	98%	59%	96%	0.69	0.62–0.76
18 months or 24 months	1374	67	22	133	1152	75%	90%	34%	98%	0.83	0.77–0.88
*Combinations of screeners*											
M-CHAT-R or ITC											
18 months or 24 months	1391	80	11	224	1076	88%	83%	26%	99%	0.85	0.81–0.89
18 months + 24 months	1514	45	43	45	1381	51%	97%	50%	97%	0.74	0.67–0.81

**TABLE 5 |:** Coefficients from logistic regressions predicting 36-month outcome (ASD or no ASD).

	Estimate	Standard error	*p*
*18 months*			
Intercept	0.45	0.27	0.096
M-CHAT-R	−1.95	0.31	< 0.001
ITC	−1.30	0.31	< 0.001
VIRSA	−1.16	0.29	< 0.001
*24 months*			
Intercept	1.95	0.40	< 0.001
M-CHAT-R	−1.95	0.38	< 0.001
ITC	−2.17	0.36	< 0.001
VIRSA	−1.71	0.39	< 0.001

## Data Availability

The data that support the findings of this study are openly available in National Database for Autism Research (NDAR) at http://ndar.nih.gov.

## Abbreviations:

ASD: autism spectrum disorder
ITC: Infant-Toddler Checklist
M-CHAT-R: Modified Checklist for Autism in Toddlers, Revised
VIRSA: Video-Referenced Infant Rating System for Autism
